# Multimodal Regulation Orchestrates Normal and Complex Disease States in the Retina

**DOI:** 10.1038/s41598-017-00788-3

**Published:** 2017-04-06

**Authors:** A. M. Olivares, A. S. Jelcick, J. Reinecke, B. Leehy, A. Haider, M. A. Morrison, L. Cheng, D. F. Chen, M. M. DeAngelis, N. B. Haider

**Affiliations:** 1 Schepens Eye Research Institute, Massachusetts Eye and Ear Infirmary, Department of Ophthalmology, Harvard Medical School, Boston, MA United States of America; 2 Genetics, Cell Biology, and Anatomy, University of Nebraska Medical Center, Omaha, Nebraska United States of America; 3 Ophthalmology and Visual Sciences, John A. Moran Eye Center, University of Utah School of Medicine, Salt Lake City, Utah United States of America

## Abstract

Regulation of biological processes occurs through complex, synergistic mechanisms. In this study, we discovered the synergistic orchestration of multiple mechanisms regulating the normal and diseased state (age related macular degeneration, AMD) in the retina. We uncovered gene networks with overlapping feedback loops that are modulated by nuclear hormone receptors (NHR), miRNAs, and epigenetic factors. We utilized a comprehensive filtering and pathway analysis strategy comparing miRNA and microarray data between three mouse models and human donor eyes (normal and AMD). The mouse models lack key NHRS (Nr2e3, RORA) or epigenetic (Ezh2) factors. Fifty-four total miRNAs were differentially expressed, potentially targeting over 150 genes in 18 major representative networks including angiogenesis, metabolism, and immunity. We identified sixty-eight genes and 5 miRNAS directly regulated by NR2E3 and/or RORA. After a comprehensive analysis, we discovered multimodal regulation by miRNA, NHRs, and epigenetic factors of three miRNAs (miR-466, miR1187, and miR-710) and two genes (Ell2 and Entpd1) that are also associated with AMD. These studies provide insight into the complex, dynamic modulation of gene networks as well as their impact on human disease, and provide novel data for the development of innovative and more effective therapeutics.

## Introduction

Regulating gene expression is a fundamental mechanism used by cells to orchestrate the complex development of all tissues. This multi-tiered event is modulated by many processes including modification of DNA, regulation of pre- and post-transcripts, and protein modifications^1–3^. Gene regulation at the DNA level occurs through several mechanisms, including chromatin modification directed by DNA methylation, and noncoding RNA (ncRNA) or DNA-binding proteins^4, 5^. Transcription factors are the main contributors in regulating networks at the transcription level. Additionally, cells regulate how much mRNA is translated into proteins by modulating capping, splicing, addition of a Poly (A) tail, the sequence-specific nuclear export rates, and by sequestering the RNA transcript^2, 6–8^. The translation of mRNA is also controlled by a number of mechanisms at initiation or by mRNA silencing. In the initiation process, recruitment of small ribosomal subunits is modulated by mRNA secondary structure, antisense RNA binding, or protein binding while the translational repression and gene silencing is modulated by microRNAs (miRNAs)^9–11^. In this study, we discover the synergistic manner in which three modulators (miRNA, nuclear hormone receptors (NHRs), and epigenetic factors) influence the retina in a normal and diseased state.

miRNAs have recently emerged as an important class of post-transcriptional regulatory factors and play a crucial role in regulating gene expression in the retina^12–14^. miRNA coding sequence typically resides in intergenic regions or on the anti-sense strand of genes^15, 16^. miRNAs are self-sufficient, retain promoters and regulatory elements, and have the capacity to regulate their own expression. Recent studies revealed that there are more than 400 miRNAs expressed in the retina, and miRNA gene regulation has been shown to affect retinal development, function, and disease^14, 17, 18^. Previous studies have demonstrated significant differences between the expression profiles of miRNAs in the embryonic and adult retina^18^. These profiles suggest specific roles for miRNAs in the developing and mature retina. Different groups and clusters of miRNAs have been identified and are usually co-expressed under similar conditions^19^. Recent studies also demonstrate that variations in gene expression involving transcription factors or miRNAs are implicated in numerous retinal diseases^14^. miRNAs play an important role in the development and maintenance of photoreceptors cells. Therefore, lack or misexpression of miRNAs are associated with diseases such as retinitis pigmentosa, age-related macular degeneration (AMD), light-induced retinal degeneration, and Stargardt disease^20–22^. Complete loss of the miR-24a, for example, impacts the rate of apoptosis in retinal precursors, leading to a decrease in the size of the eye^23^. Global loss of retinal miRNAs by loss of Dicer expression has severe and detrimental effects on retinal development and physiology. Several loss of function studies reveal miRNAs are crucial regulators of differentiation and promote the survival of retinal neuronal progenitors^14, 24, 25^. In addition, altered miRNA expression has been demonstrated in rodent models of Retinitis Pigmentosa (RP), Induced Uveitis, Retinoblastoma, and Diabetic Retinopathy^26–28^. Consistent with these studies, retinas have a different expression miRNA profile than normal retinas^29^. Furthermore, it is increasingly evident that the two classes of gene modulators, transcription factors and miRNAs, work synergistically within given gene networks^30–32^.

Nuclear hormone receptors (NHRs) are part of a large superfamily of ligand-dependent steroid hormone receptors that often regulate transcription in a ligand-dependent manner and can function as activators or repressors of gene expression. NHRs regulate many biological processes to maintain cellular homeostasis and are significant regulators of retinal development, maintenance, and disease^33–37^. miRNAs are regulated by nuclear receptors via three main mechanisms: binding to the promoter region, indirect regulation by interacting with nuclear receptor target genes, and regulation of miRNA biogenesis^38^. Nuclear receptor class 2, subfamily e, member 3 (Nr2e3) and retinoic acid receptor-related orphan receptor alpha (Rora) are key regulators of retinal development and function^33, 39–45^. Mutations in *NR2E3* are associated with several retinal diseases including enhanced S-cone syndrome, Goldman-Favre Syndrome, Clumped Pigmentary Retinal Degeneration, and Autosomal Recessive and Dominant Retinitis Pigmentosa^46^. Mice lacking *Nr2e3*, *Nr2e3* ^*rd7*/*rd7*^, *rd7*, exhibit a slow progressive retinal degeneration, characterized clinically by pan-retinal spotting, and histologically by whorls and rosettes due to excess production of blue opsin expressing cone cells followed by progressive degeneration of both rod and cone photoreceptor cells^41, 47, 48^.

Our prior studies show *Nr2e3* regulates *Rora* ^42^ and both are implicated in AMD pathogenesis^49–51^. RORA is ubiquitously expressed and plays a role in lipid metabolism, bone morphogenesis, and photoreceptor development^50^. Studies of the knockout mouse model for *Rora* (Staggerer) show it is highly expressed in cerebellar Purkinje cells, and plays a crucial role in the CNS^51^. Staggerer mice are a model for human ataxia characterized by reduced number of Purkinje cells^52, 53^. In the retina, *Rora* is expressed in the ganglion cell layer and the inner nuclear layer of the adult retina^54, 55^, and regulates the expression of the cone opsin genes, *Opn1sw* and *Opn1mw* ^45^. More recently, our colleagues discovered *Nr2e3* is upregulated in the conditional knockout of epigenetic factor Enhancer Of Zeste 2 Polycomb Repressive Complex 2 Subunit, Ezh2, suggesting a link between *Nr2e3* and epigenetics^54^.

In addition to miRNA and NHR regulation, epigenetic events such as DNA methylation and post-translational modifications of the histones regulate gene expression. The marker histone-3, on lysine-27 (H3K27me3), trimethylates histones is a strong epigenetic regulator that serves to silence genes and is regulated by EZH2^56^. EZH2 is a subunit of the Polycomb Repressor Complex2 (PRC2) and is involved in the regulation of a key development transcription factor family of genes including homeobox (HOX), SRY box (SOX), paired box (PAX) and wingless type (WNT). EZH2 functions in the PCR2 histone methyltransferase complex involved in the silencing of HOX genes during development^57^. In the PCR2 complex, EZH2 acts as a catalytic agent that methylates lysine 9 and 27 in histone H3^56, 58^. EZH2 as well as other genes from the PCR2 complex induce the overexpression of E2F, a cell cycle regulator, in tumor cell lines^59^. EZH2 also interacts with retinoic acid (RA) to induce neural stem cell differentiation^59–61^. Interestingly, microarray data of *Ezh2* ^*−*/*−*^ retinas detected upregulation of *Nr2e3* consistent with a phenotype of suppression of neural proliferation and differentiation^62^. *Ezh2* ^*−*/*−*^ mice exhibit retinal degeneration characterized by functional loss of photoreceptors, detected by loss of scotopic and photopic ERG responses, and histologically by retinal thinning at one month of age.

Macular degeneration is a leading cause of blindness yet there are no known genetic causes, and therapies are not effective on a significant number of patients. It is clear from many studies that gene expression and related disease occur via a dynamic and complex synergistic integration of multiple mechanisms of regulation. In this study, we examine the synergistic modulation of NHRs *Nr2e3* and *Rora*, miRNAs, and the epigenetic factor *Ezh2*. As both *NR2E3* and *RORA* have been implicated in AMD, we evaluated our miRNA potential gene targets as well as differentially expressed genes from each mouse model against gene expression data from a large cohort of AMD patients’ donor eyes from unrelated subjects. Lastly, we determined which of these differentially expressed genes and miRNAs are directly regulated by NR2E3 and/or RORA. This is the first comprehensive study to evaluate multiple mechanisms of gene regulation and determine their combined impact on biological pathways and human disease.

## Results

### The retina has progressive, dynamic miRNA expression

**Figure 1:**
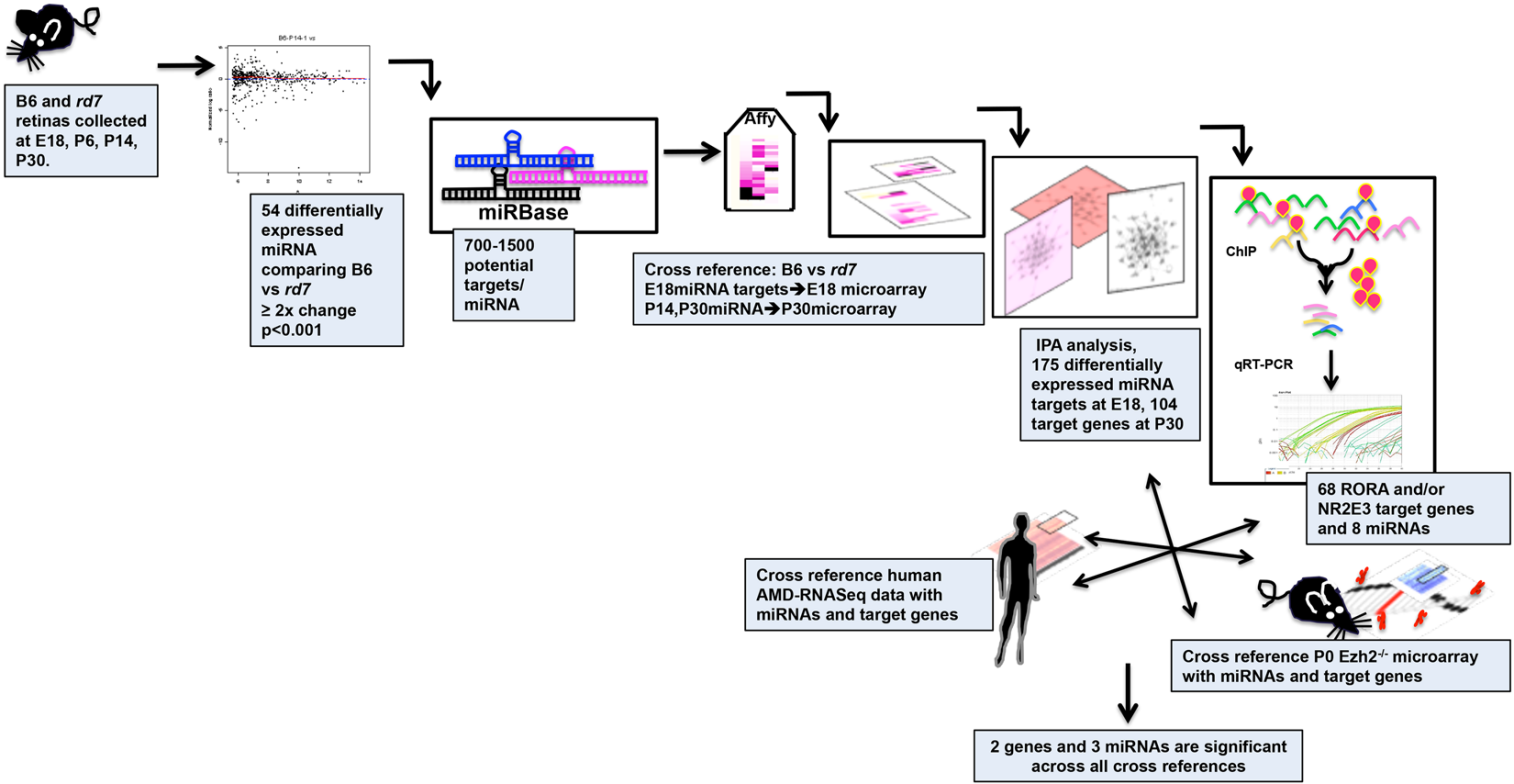
Comprehensive filtering strategy to identify multimodal regulation of miRNAs and their target genes. Step 1. 10 retinas per time point (E18, P6, P14, P30) were collected from B6 and *rd7*. Step 2. miRNA microarray was performed on samples from step 1. 54 differentially miRNAS were identified with 2-fold change, P < 0.001. Step 3. Subsequent filtering analysis of miRNA expression data was performed using BRB Array Tools along with Stanford Statistical Analysis of Microarrays (SAM) and evaluated using miRBase to identify their potential target genes. Step 4. miRNA targets were then cross-referenced with E18 microarray and P30 *rd7* microarray data. Steps 5, 6. Genes that were found to be statistically significant were analyzed using Ingenuity Pathway analysis to algorithmically generated gene networks in which the default cutoffs identify those genes that were significantly differentially regulated in the network. Step 7. Genes that were identified through the networks were screened for the nuclear receptor-binding site of Nr2e3 and RORA and further analyzed by chromatin immunoprecipitation. A total of 68 genes were direct targets of RORA and/or Nr2e3 as well as 8 miRNAs (out of the 54 differentially expressed miRNAs identified). The results obtained were then cross-referenced to AMD-RNAseq data and microarray data from the epigenetic factor Ezh2. This filtering strategy allowed us to identify 2 genes (Ell2 and Entpd1) as well as 3 miRNAs (mir466, miR1187 and miR710) that are regulated by epigenetic factors and nuclear hormone receptors and are associated with AMD pathogenesis.

**Figure 2:**
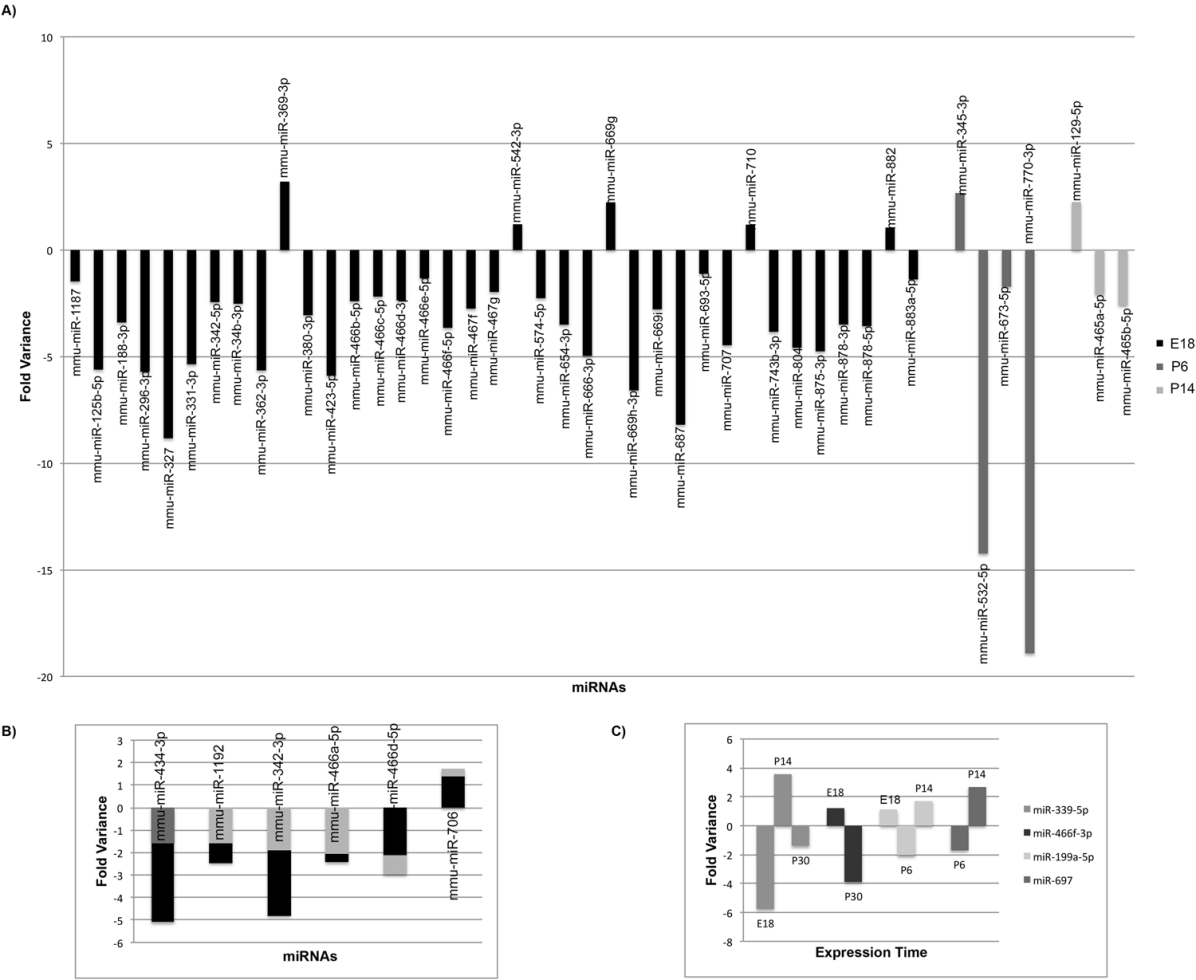
miRNAs differentially expressed between B6 (wildtype) and *rd7* (mutant) retinas. (**A**) miRNAs that are differentially expressed at E18, P6, or P14 (**B**) miRNAs differentially expressed between the embryonic time point and one adult time point, and (**C**) miRNAs differentially expressed in more that one time point and have an alternate pattern of expression. Minimum 2 fold change and P < 0.001.

In this study, we employed a comprehensive analysis and filtering strategy to identify key miRNA and their target genes that are differentially expressed in *rd7*, regulated by *Rora*, *Nr2e3*, and *Ezh2*, and implicated in AMD (Fig. 1). A miRNA microarray analysis was conducted on retinas from *Nr2e3* ^*rd7*/*rd7*^ and control C57BL6/J mice at embryonic (E) 18.5 day and postnatal (P) days 6, 14, and 30 to capture the dynamic changes that occur in a developing retina to its maturity. Differential miRNA expression was observed across multiple time points, (Supplemental Figure S1). Interestingly, this heat map shows clear differences in the expression of miRNAs between the embryonic and adult points and postnatal day 6 (P6) illustrates a transition between development and maturity. In the initial analysis and filtering, redundant probes, erroneous control spots, and miRNAs with no known gene targets were removed from analysis and miRNAs differentially expressed in more than one time point were classified as “shared”. Using these criteria, 54 miRNAs were differentially expressed between *Nr2e3* ^*rd7*/*rd7*^ and *C57BL6*/*J* mice with a minimum 2-fold change and a p value of p < 0.001. Of the differentially expressed miRNAs, 37 miRNAs were unique to E18, 4 miRNAs were unique to P6, 3 miRNAs were unique to P14, and 10 miRNAs were shared between embryonic time point and one or multiple postnatal time points (Fig. 2A,B). Interestingly, while most of the miRNA that were differentially expressed at multiple time points were either consistently increased or decreased, four miRNAS showed altered pattern of expression at each time point they were differentially expressed (Fig. 2C). Previous studies of miRNA profiles expressed in the embryonic and adult retina were cross-referenced revealing common and unique miRNAs to our study^17, 18^. Additionally, prior studies from various models of retinal degeneration identified over 300 differentially expressed miRNAs^63–90^, a total of 16 common miRNAs were identified (miR-1187, miR-125b-5p, miR-331-3p, miR466d-3p, miR-467f, miR-542-3p, miR-574-5p, miR654-3p, miR669h-3p, miR-882, miR-342-3p, miR-466a-5p, miR-466d-5p, miR-706, miR-345-3p, miR532-5p). This study reveals 38 unique miRNAs that may be particular to the Nr2e3 regulated pathway. All 54 differentially expressed miRNAs were subsequently evaluated against the mirBase to determine all potential target genes for each miRNA (Table 1). Each miRNA can have up to 700–1500 potential target genes. Potential miRNA target genes were cross-referenced to previously published microarray expression data (GSE24512) of genes misexpressed in *Nr2e3* ^*rd7*/*rd7*^ retinas at E18 and P30.

**Table 1:** miRNAs differentially expressed in *rd7* vs B6 and their potential target genes.

Expression TP	miRNA	Target Genes
E18	miR-1187	Abcc9, Acpp, B3gnt9, Bnc2, Casp12, Cpm, Crhbp, Dpt, Egfl6, Entpd1, Fndc1, Fras1, Has2, Lcp1, Mylk4, Nox4, Otx1, Papss2, Peg3, Pgm5, Rgs5, Stard8, Stat6, Tek, Tfcp2l1, Vamp5, Wnt4
	miR-125b-5p	Acer2, Atp1b4, Bmf, C77080, Ehd4, Enpep, Ets1, Eva1a, Hapln1, Jade2, Mgll, Msrb3, Rhov, Serpinb5, Snx31, Ucp2
	miR-188-3p	Alcam, Arhgef3, Atp1b4, Cd248, Cxcl12, Egln3, Ell2, Flrt2, Gbp7, Grhl2, Ifi202b, Itgb3, Itpripl2, Msrb3, Muc4, Myot, Scara5, Slc16a12, Smoc2
	miR-296-3p	Epb4.1l1
	miR-327	Abca9, Arhgap36, Col18a1, Crim1, Cspg4, Cxcl12, Entpd1, Fbn2, Iigp1, Kcnq5, Lamp2, Ppfibp2, Ptprb, Ryr1, Thbs1, Tmem88
	miR-331-3p	Mgll
	miR-342-5p	Ppargc1a
	miR-34b-3p	Fras1
	miR-362-3p	Il13ra1, Mef2c, Ppp1r3b, Prdm16, Sspn, Unc45b
	miR-369-3p	Afap1l2, Aspn, Mylk4, Sema3d, She
	miR-380-3p	Dst
	miR-423-5p	Bmf, Ccnd2, Dlk1, Mgll, Myrf, Slc7a8
	miR-466b-5p	Acer2, Fam46a, Ppargc1a, Tbx22
	miR-466c-5p	Acer2, Fam46a, Ppargc1a, Tbx22
	miR-466d-3p	Arap2, B3gnt9, Dmrta2, Dsg2, Dst, Ets1, Fam46a, Fgfr2, Gdf10, Jag1, Matn2, Piezo2, Sgms1, Stard8, Tbx22, Tgfbr3
	miR-466e-5p	Abcc9, Acpp, B3gnt9, Bnc2, Casp12, Crhbp, Dpt, Egfl6, Entpd1, Fndc1, Fras1, Has2, Lcp1, Mylk4, Nox4, Otx1, Papss2, Peg3, Pgm5, Rgs5, Stard8, Stat6, Tek, Tfcp2l1, Vamp5, Wnt4
	miR-466f-5p	Fzd4
	miR-467f	Dmrta2, Fam46a, Fam84a, Fndc1, Hmcn1, Itpripl2, Jag1, Mef2c, Msrb3, Stard8, Tbx22, Tek, Thbs2, Trp63
	miR-467g	Arap2, B3gnt9, Dmrta2, Dsg2, Dst, Ets1, Fam46a, Fgfr2, Fgl2, Gdf10, Jag1, Matn2, Piezo2, Sgms1, Stard8, Tbx22, Tgfbr3
	miR-542-3p	Cgnl1, Fgl2, Jade2, Lpp, Ppp1r3b, Slc7a8, Sspn, Tgfbr3
	miR-574-5p	Acpp, Fndc1, Mylk4, Ntrk2, Rgs5, Vcan
	miR-654-3p	Cd55, Lamc1, Ntrk2, Ppp1r3b, Thbs2
	miR-666-3p	Bmp7, C77080, Ets1, Fam174b, Fzd4, Ptgfrn, Rab27a
	miR-669g	Ntrk2
	miR-669h-3p	Dsc2, Fndc1, Foxc1, Frk, Tbx22
	miR-669i	Cd36, Fgl2, Fndc1, Gatm, Tbx22
	miR-687	Crim1, Dsg2, Mfsd7c, Msrb3
	miR-693-5p	Egfl6, Foxc1, Il13ra1, Mef2c, Papss2
	miR-707	Ehf
	miR-710	Dsg2, Jag1, Lpar1, Mef2c
	miR-743b-3p	Acer2, Afap1l2, Bnc2, Cast, Ell2, Elovl7, Flrt2, Fndc3b, Lpp, Lrig1, Npr3, Ntrk2, Ptgfrn, Scara5, Sema3c, Tgfbr2
	miR-804	B3gnt9, Dpt, Foxc1, Osr1
	miR-875-3p	Bnc2, Cd44, Ehf, Fam46a, Fgl2, Frk, Il13ra1, Lamc1, Mef2c, Otx1, Ptgfrn, Rbms1, Sema3c, Sgms2, Sspn, Tfcp2l1
	miR-878-3p	Cgnl1, Elk3, Mef2c, Sema3c, Sema3d
	miR-878-5p	Ptprb, Trdn
	miR-882	Atp1b4, Bmf, Dsc2, Fosl2, Mgll, Ntrk2, Rgs5, Sgms1, Tfcp2l1
	miR-883a-5p	Bnc2, Eef2k, Fam174b, Lyz1
E18, P6	miR-434-3p	Sulf1
E18, P14	miR-1192	Bmp7, Boc, Ccbe1, Ccnd2, Cd44, Cd55, Col25a1, Dennd2c, Dmp1, Ehf, Ell2, Elovl7, Ets1, Fam101b, Fbn2, Flt1, Fosl2b, Foxc1, Fzd6, Hapln1, Itpripl2, Kdelc2, Lmo7, Lpp, Mef2c, Mylk4, Nrp2, Ntrk2, Pdlim7, Piezo2, Prickle1, Sema3c, Sgms1Sgms2, Slc7a8, Tbx18, Tgfbr3, Thbs2, Trp63, Unc45
	miR-342-3p	Ntrk2
	miR-466a-5p	Abcc9, Acpp, B3gnt9, Bnc2, Casp12, Crhbp, Dpt, Egfl6, Entpd1, Fndc1, Fras1, Has2, Lcp1, Mylk4, Nox4, Otx1, Papss2, Peg3, Pgm5, Rgs5, Stard8, Stat6, Tek, Tfcp2l1, Vamp5, Wnt4
	miR-466d-5p	Abcc9, Acpp, B3gnt9, Bmp7, Bnc2, Ccbe1, Cpm, Eef2k, Fam114a1, Fam174b, Fam84a, Has2, Lpp, Msrb3, Nrk, Osr1, Otx1, Papss2, Peg3, Pgm5, Rgs5, Slc7a8, Sspn, Stard8, Stat6, Sulf1, Vamp5, Wnt4
	miR-706	Atp1b4, Dpt, Has2, Il13ra1, Kdelc2, Mef2c, Mylk4, Ostf1, Sema3d, Sgms2
E18, P30	miR-466f-3p	Adora2b, Col25a1, Fam174b, Fam46a, Fam84a, Fbn2, Fndc1, Hmcn1, Itpripl2, Jag1, Lpar1, Macc1, Matn2, Mef2c, Msrb3, Mylk4, Ntrk2, Otx1, Pdgfc, Sspn, Stard8, Tfcp2l1, Thbs2, Trp63
E18, P6, P14	miR-199a-5p	Afap1l2, Cd36, Cgnl1, Ctgf, Ets1, Fzd4, Fzd6, Hapln1, Hmcn1, Itga8, Lamc1, Lrrc173, Matn2, Mertk, Myrf, Ppargc1a, Scara, Sulf1, Zc3hav1
E18, P14, P30	miR-339-5p	Kdelc2, Lamc1, Sema3c, Sspn, Tfcp2l1
P6	miR-345-3p	Cpm, Eef2k
	miR-532-5p	Alcam, Ets1, Gbp7, Itpripl2, Lpp, Ppp1r3b
	miR-770-3p	Hapln1, Ucp2
	miR-673-5p	Abca1, Fzd4, Kdelc2, Nrk, Tfcp2l1
P6, P14	miR-697	Lamp2, Flt1, Rbsm1
P14	miR-129-5p	Flt1, Stat6
	miR-465a-5p	Abcc9, Acpp, B3gnt9, Bnc2, Casp12, Crhbp, Dpt, Egfl6, Entpd1, Fndc1, Fras1, Has2, Lcp1, Mylk4, Nox4, Otx1, Papss2, Peg3, Pgm5, Rgs5, Stard8, Stat6, Tek, Tfcp2l1, Vamp5, Wnt4
	miR-465b-5p	Acer2, Fam46a, Ppargc1a, Tbx22

Genes identified through microarray data of differentially expressed genes in *rd7* vs B6. Minimum 2 fold change and p < 0.05.

### Identification of microRNA target genes and their gene networks

**Figure 3:**
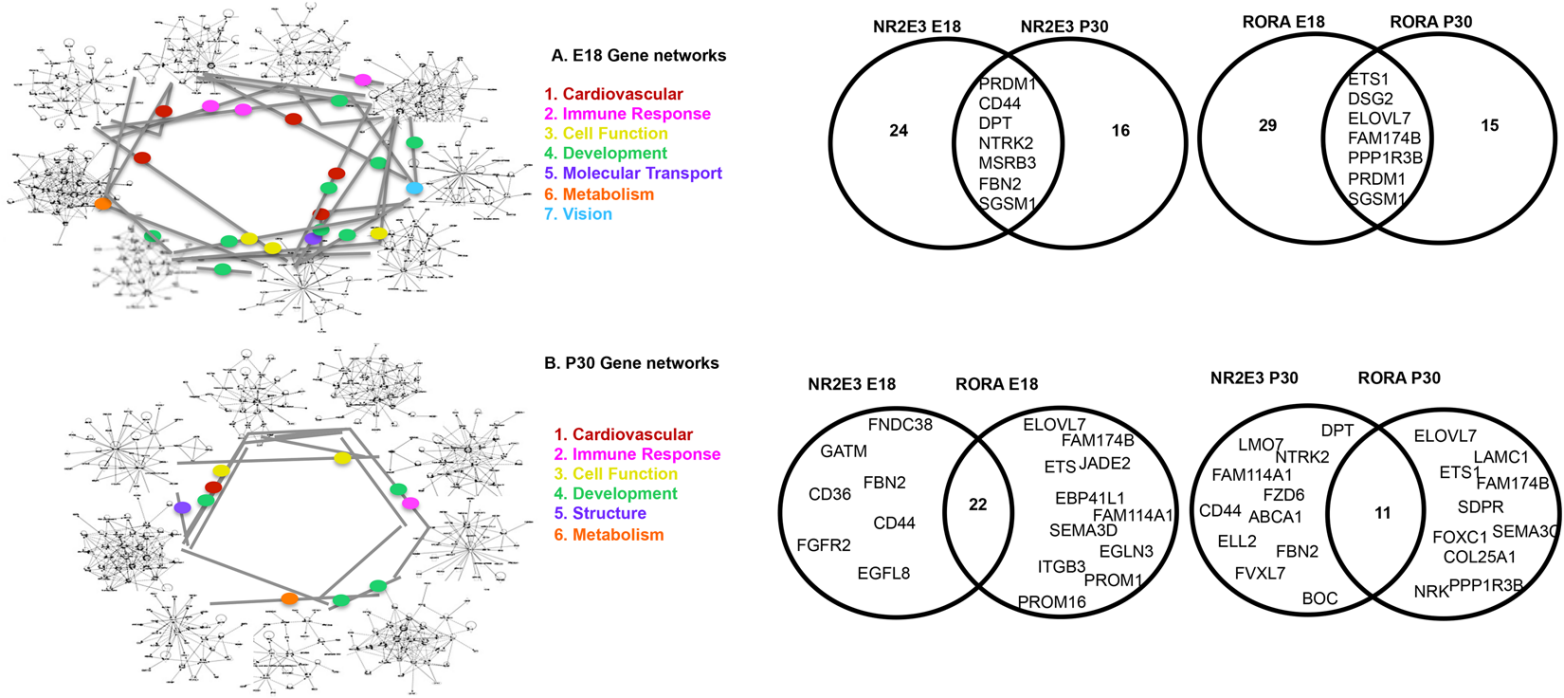
Interacting NR2E3 and RORA Associated Gene Networks. (**A**) IPA analysis of E18 targets identified 9 gene networks with 7 biological classifications. (**B**) IPA analysis of P30 targets identified 9 gene networks with 6 biological classifications. Venn Diagrams show unique and overlapping gene targets of NR2E3 and RORA at E18 and P30. Comparisons of RORA E18/P30 or NR2E3 E18/P30 show less overlap than RORA/NR2E3 at E18 or RORA/NR2E3 at P30.

Once significantly differentiated miRNA were identified, the analysis focused on miRNAs differentially expressed at embryonic day E18.5 and postnatal day P30.5 for further analysis. These time points were chosen to represent miRNAs important in development when *Nr2e3* is first expressed, and those miRNAs important to maintain normal function in the mature retina. Potential miRNA target genes at each of the two time points were then cross-referenced to microarray expression data^91^. To identify differentially expressed miRNA potential target genes showing biological significance, miRNA target genes were cross-referenced with microarray data mentioned above (GSE24512) and those exhibiting greater than 2-fold change of gene expression in *Nr2e3* ^*rd7*/*rd7*^ when compared to *C57BL6*/*J* mice were further analyzed. Duplicate genes (targeted by more than one miRNA within a time point) were counted once in the total number of unique miRNA target genes. This filtering strategy identified over 150 unique genes (Supplemental Table S1) which were further analyzed by Ingenuity Pathway Analysis (IPA). IPA analysis revealed eighteen unique gene networks including those for metabolism, development, immune response, cancer, and cellular function (Table 2). These putative miRNA targets are associated with over 50 diseases including diabetes, mellitus, cell proliferation of cancer, neuro-inflammation, apoptosis of neuroblastomas, and heart disease (Table 3). Figure 3 summarizes the combined networks and their common targets and pathways.

**Table 2:** Ingenuity pathway analysis (IPA) of miRNA, RORA and NR2E3 target genes.

E18 Network Function	Genes
Cardiovascular	ANTXR2, ARHGEF3, ASPN, B3GNT9, BMP7, ***CAST***, *DSC2*, **DSG2**, *EGLN3*, *EHF*, ***ELL2***, *ETSS1*, FAM46A, **FGFR2**, FOXC1, **GATM**, GRHL2, HAS2, **JAG1**, LAMC1, LAMP2, LRIG1, ***MGLL***, MUC4, ***NTRK2***, OSR1, PPARGC1A, *PRDM16*, ***PTPRB***, RSPO1, RYR1, ***SEMA3C***, *SEMA3D*, ***SGMS1***, SLC7A8, TGFBR2, TGFBR3, THBS1, THBS2, TRDN, WNT4
Immune Response/Injury	***CD44***, ***COL18A1***, ***DPT***, **EGFL6**, ELK3, ENTPD1, FGL2, FOSL2, ***FRAS1***, KCNQ5, ***LPP***, LYZ, MYRF, NPR3, OTX1, RAB27A, RGS5, VCAN
Cellular Function	***ALCAM***, ARAP2, BACH2, ***BMF***, BNC2, CCND2, **CD36**, CRHBP, CSPG4, DLK1, DST, *ELOVL7*, FLRT2, ***IFI202B***, IL13RA1, *ITGB3*, LCP1, MYLK4, PPFIBP2, ***PPP1R3B***, ***PRDM1***, SCARA5, SGMS2, **SHE**, STAT6, TBX22, TEK, TFCP2L1, UNC45B
Development	ACER2, *CGNL1*, ***CRIM1***, DMRTA2, *EPB41L1*, **FBN2**, **FNDC3B**, FOXC1, FZD4, GBP7, *JADE2*, **JAG1**, ***LPAR1***, MATN2, MEF2C, ***MSRB3***, MYOT, ***PAPSS2***, PEG3, PGM5, RBSM1, SLC16A12, SMOC2, SSPN, STARD8, TRP63, VAMP5
Molecular Transport	ABCA9, CD248, *FAM174B*, FAM84A, FNDC1, ITPRIPL2, ***LRFN2***, PIEZO2, PRTG
Metabolism	AFAP1L2, ARHGAP36, CXCL12, EEF2K, **FRK**, *HMCN1*, NOX4, PTGFRN
Vision	***CGNL1***, ***CRIM1***, DMRTA2, *EPB41L1*, **FBN2**, **FNDC3B**, FZD4, KIAA1522, MATN2, ***MSRB3***, ***PAPSS2***, PGM5, RBSM1, SLC16A12, STARD8, VAMP5
**PN Network Function**	**Genes**
Cardiovascular	***ARG1***, BACH2, BMP7, **BOC**, **CAV3**, ***CD44***, *COL25A1*, DLK1, ELK3, ETS1, **FLT1**, ***FOXC1***, ***HAPLN1***, HAS2, **JAG1**, *LAMC1*, LCP1, **LPP**, NOX4, NRP2, **NTRK2**, OSTF1, RSPO1, ***SEMA3C***, ***SEMA3D***, SLC7A8, SULF1, TEK, TGFBR3, THBS2, VCAN, WNT4
Structure	***ABCA1***, ABCC9, B3GNT9, CASP12, *CCBE1*, CCND2, ***CD55***, **CD93**, *EHF*, ***ELL2***, FAM46A, IL13RA1, OSR1, *PPARGC1A*, *PRDM1*, STAT6, UCP2
Hematological Disease	FAM101B, ***FAM114A1***, ***FAM174B***, ***FAM84A***, ***FZD6***, KDELC2, ***MSRB3***, PAPSS2, PDLIM7, PIEZO2, ***PRICKLE1***, SGMS2, TMEM164, VAMP5
Cellular Function	ACPP, **DPT**, EEF2K, EGFL6, EHD4, ***ENTPD1***, FOSL2, FRAS1, ***LMO7***, LYZ1, MYRF, OTX1, RGS5, SDPR, TNNT2
Immune Response/Injury	ASPN, BNC2, *CPM*, DENND2C, ***FBN2***, FIBIN, FNDC1, ITPRIPL2, NEBL, ***PPP1R3B***, STARD8, TBX18
Metabolism	CRHBP, DEK, DMRTA2, ***FBXL7***, *GATM*, *NRK*, RBSM1, ***SGMS1***, SMPX, TFCP2L1, DST, *ELOVL7*, MYLK4, PGM5, TBX22, UNC45B, SLC7A10
Development	ACER2, *DSG2*, ***EGFR***, FZD4, LMNA, MEF2C, PEG3, SSPN, TRP63

Genes regulated by NR2E3 are in bold; genes regulated by RORA are in italics, and genes regulated by NR2E3 and RORA are bold and italics.

**Table 3:** Ingenuity pathway analysis (IPA) of miRNA differentially expressed between *rd7* and B6.

	miRNA	Disease Annotation
Cancer	miR-185-5p, miR-199a-5p	Early invasive cervical squamous cell carcinoma
	miR-542-3p	Cell proliferation of bone cancer cell lines
	miR-542-3p	Cell proliferation of ovarian cancer cell lines
	miR-542-3p	Cell proliferation of brain cancer cell lines
	miR-125b-5p	Colony formation of bladder cancer cell lines
	miR-125b-5p, miR-185-5p, miR-199a-5p miR-331-3p, miR-542-3p	Proliferation of tumor cell lines
	miR-125b-5p	Advanced Dukes’ stage colorectal cancer
	miR-185-5p, miR-199a-5p	Proliferation of cervical cancer cell lines
	miR-125b-5p, miR-185-5p, miR-199a-5p	Squamous-cell carcinoma
	miR-125b-5p	Apoptosis of neuroblastoma cells
	miR-125b-5p	Metastatic HER2 negative hormone receptor
	miR-125b-5p, miR-339-5p, miR-542-3p	Hematologic cancer
	miR-125b-5p	Apoptosis of mammary tumor cells
	miR-125b-5p, miR-199a-5p, miR-329-3p miR-331-3p, miR-423-5p, miR-495-3p, miR-542-3p, miR-574-5p, miR-654-3p	Colorectal cancer
	miR-185-5p	Arrest in G1 phase of colon cancer cell lines
	miR-185-5p, miR-199a-5p	Renal clear cell adenocarcinoma
	miR-339-5p	Precursor B-cell acute lymphoblastic leukemia
	miR-125b-5p, miR-331-3p	Proliferation of prostate cancer cell lines
	miR-125b-5p	Differentiation of embryonic stem cell lines
	miR-542-3p	Cell proliferation of adenocarcinoma cell lines
	miR-125b-5p, miR-339-5p	Lymphoproliferative malignancy
	miR-199a-5p	Response of endothelial cell lines
	miR-125b-5p	Migration of microvascular endothelial cells
Diabetes	miR-125b-5p, miR-327, miR-434-3p	Non-insulin-dependent diabetes mellitus
	miR-125b-5p, miR-185-5p, miR-327 miR-329-3p, miR-434-3p	Diabetes mellitus
Cardiovascular	miR-199a-5p, miR-423-5p	Failure of heart
	miR-199a-5p	Interstitial fibrosis of heart
	miR-125b-5p*, miR-199a-5p	Fibrosis of heart
	miR-199a-5p	Vasoconstriction of aorta
	miR-199a-5p	Enlargement of heart
Inflammation	miR-125b-5p, miR-185-5p, miR-188-3p miR-199a-5p, miR-423-5p	Inflammation of body region
	miR-188-3p	Inflammation of hippocampus
	miR-125b-5p, miR-185-5p, miR-199a-5p miR-423-5p	Inflammation of body cavity
	miR-125b-5p, miR-185-5p, miR-188-3p miR-199a-5p, miR-423-5p	Inflammation of organ
	miR-188-3p	Neuroinflammation
	miR-125b-5p	Differentiation of granulocyte progenitors
	miR-125b-5p	Transendothelial migration of monocytes
	miR-125b-5p	Activation GM-CSF dependent marrow macrophage
	miR-125b-5p	M1 polarization of M2 macrophages
Disease	miR-125b-5p, miR-423-5p	Rapidly progressive idiopathic pulmonary fibrosis
	miR-125b-5p	Myelodysplastic syndrome with 5q- syndrome
	miR-125b-5p	Formation of tubules
	miR-199a-5p	Chronic hepatitis B
	miR-125b-5p, miR-199a-5p, miR-423-5p	Fibrosis
	miR-199a-5p, miR-339-5p, miR-495-3p	Nonobstructive azoospermia
	miR-329-3p	Dent’s disease
	miR-125b-5p	Systemic sclerosis
	miR-125b-5p, miR-185-5p	Chromosomal aberration
	miR-125b-5p	Androgenic alopecia
	miR-185-5p	DiGeorge syndrome

Five major groups with a P value p < 0.05 were identified with the majority of the miRNAs associated with cancer.

### Nr2e3 and Rora co-regulate AMD pathways with miRNAS

**Figure 4:**
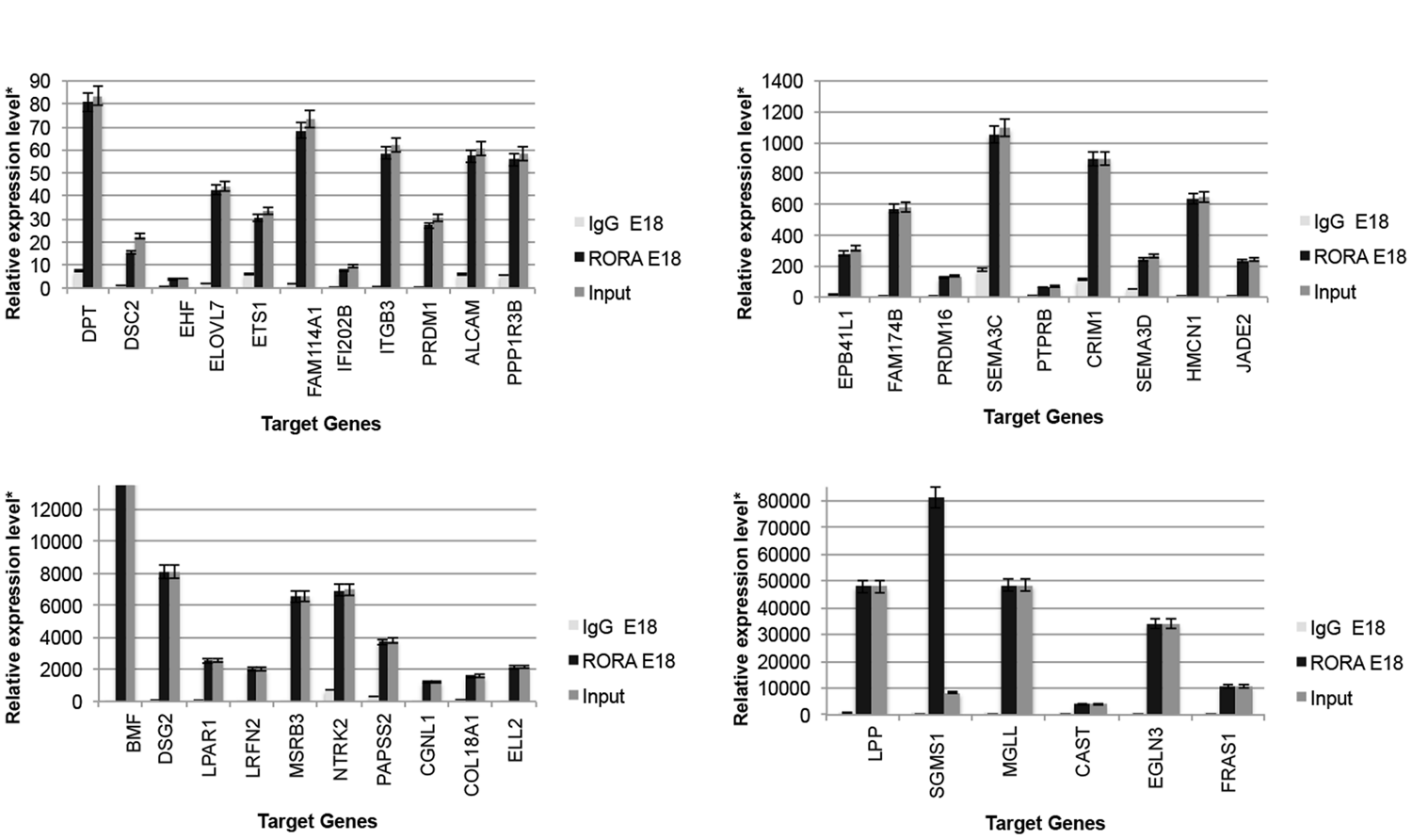
Chromatin IP (chIP) quantitative real time PCR (chIP-qRT-PCR) of gene targets for RORA at E18. Input (positive control), RORA-chIP, and immunoglobulin G (IgG) negative control. *Relative expression is calculated as molecules/1000 molecules β-actin. Standard errors are indicated for each gene. All genes had a minimum significance of p < 0.05.

**Figure 5:**
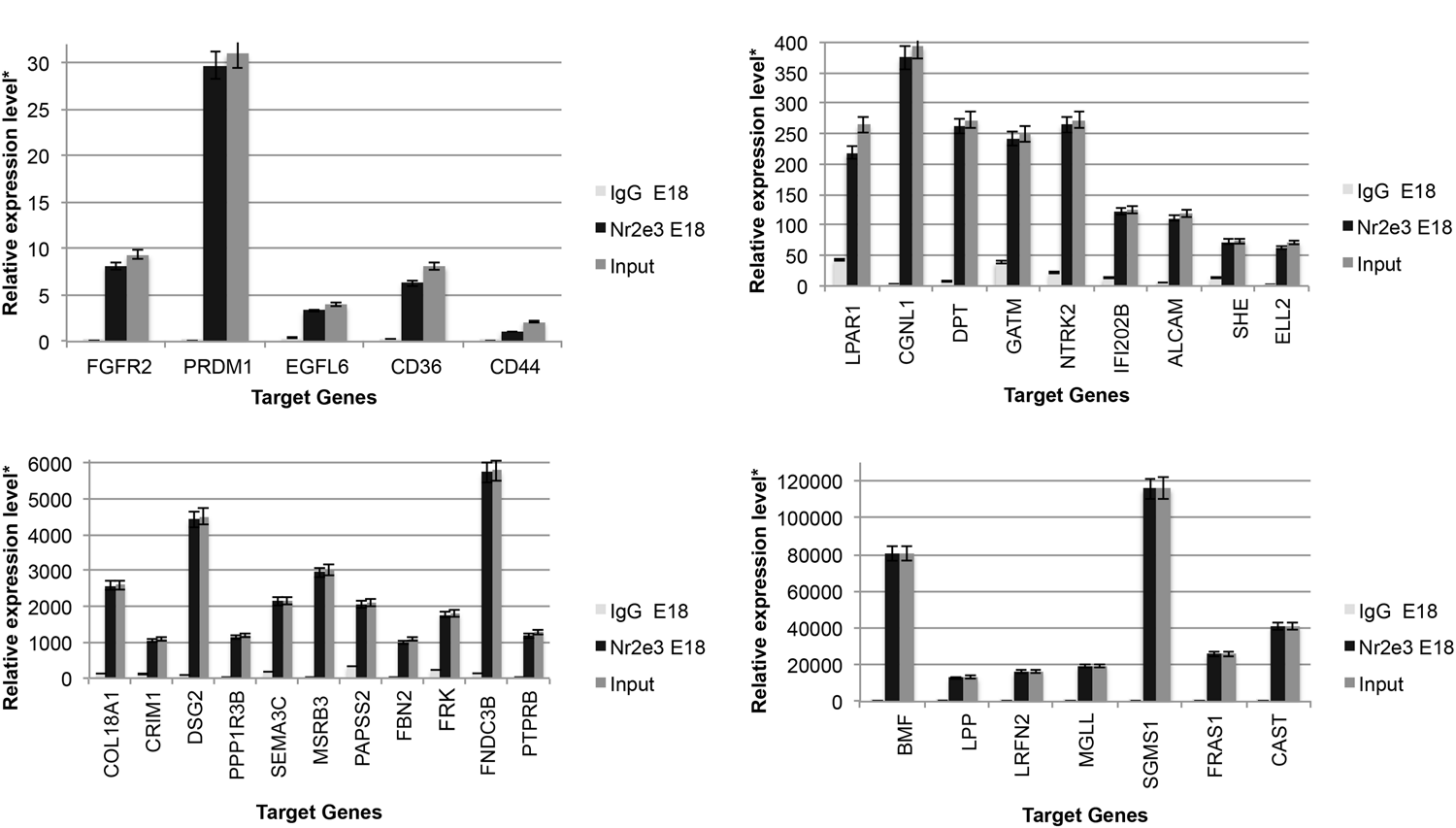
Chromatin IP (chIP) quantitative real time PCR (chIP-qRT-PCR) of gene targets for NR2E3 at E18. Input (positive control), NR2E3-chIP, and immunoglobulin G (IgG) negative control. *Relative expression is calculated as molecules/1000 molecules β-actin. Standard errors are indicated for each gene. All genes had a minimum significance of p < 0.05.

**Figure 6:**
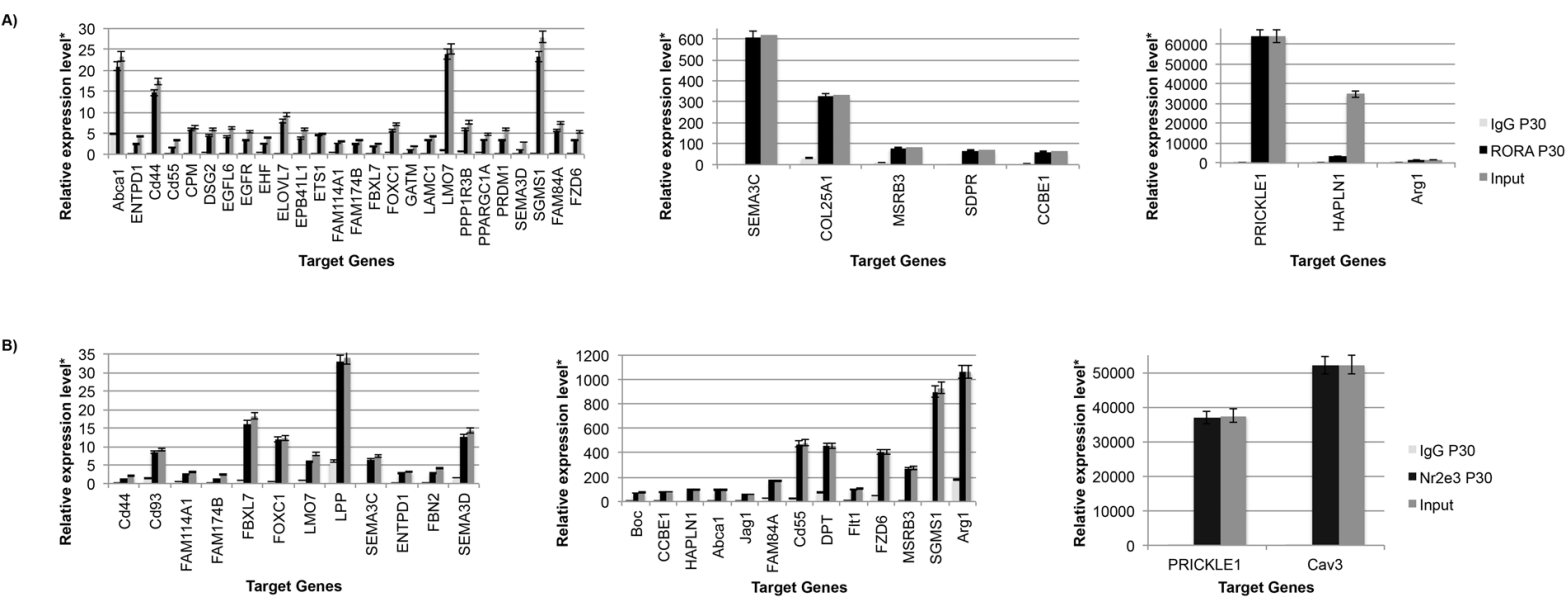
Chromatin IP (chIP) quantitative real time PCR (chIP-qRT-PCR) of gene targets for RORA and NR2E3 at P30. Input (positive control), RORA-chIP, NR2E3-chIP, and immunoglobulin G (IgG) negative control. *Relative expression is calculated as molecules/1000 molecules β-actin. Standard errors are indicated for each gene. All genes had a minimum significance of p < 0.05.

**Figure 7:**
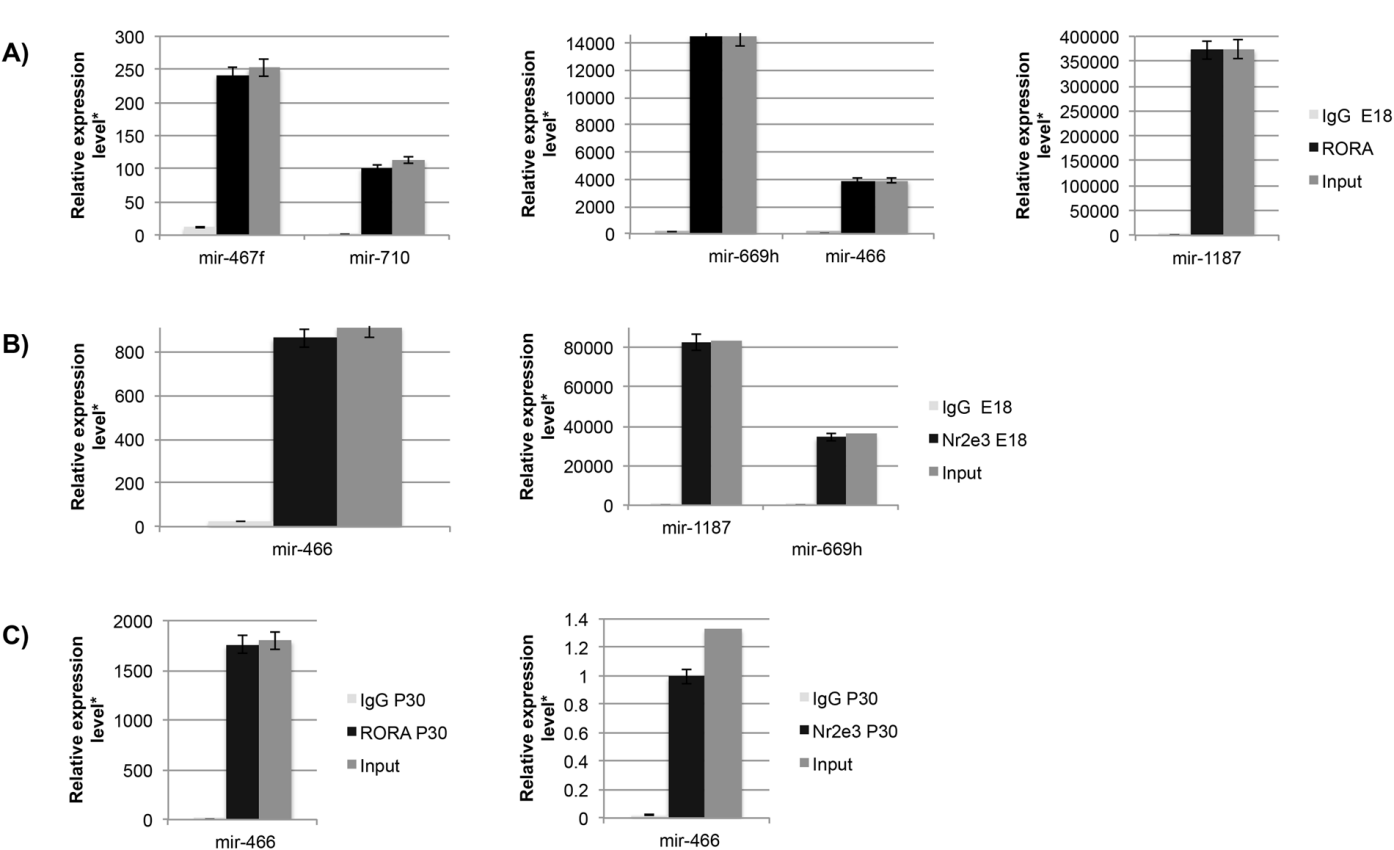
Chromatin IP (chIP) quantitative real time PCR (chIP-qRT-PCR) of miRNA targets for RORA and NR2E3 at E18 and P30. (**A**) RORA targeted miRNAs identified at E18 (**B**) NR2E3 targeted miRNAs identified at E18, (**C**) RORA and NR2E3 targeted miRNAs identified at E18. Input (positive control), RORA-chIP, NR2E3-chIP, and immunoglobulin G (IgG) negative control. *Relative expression is calculated as molecules/1000 molecules β-actin. Standard errors are indicated for each gene. All genes had a minimum significance of p < 0.05.

Previous work from our lab and others has demonstrated that the nuclear hormone receptors *Nr2e3* and *Rora* are key regulators of retinal development and function^41–45^. To determine which miRNA potential target genes and which miRNAs are directly regulated by NR2E3 or RORA, we scanned approximately 100 kilobases (kb) upstream and into intron 1 of each target gene for putative response element (RE) binding sites. We identified 87 genes and 10 miRNAs that had putative Nr2e3 and/or RORA RE binding sites. Chromatin immunoprecipitation (ChIP) revealed 68 direct target genes of NR2E3 or RORA in the developing (E18) and/or mature retina (P30) (Figs 4–6) as well as 7 miRNAs (Fig. 7). At E18.5 there were 36 target genes identified for RORA (Fig. 4) and 32 for NR2E3 (Fig. 5) with 23 of these genes common targets for both RORA and NR2E3. At P30, we identified 34 RORA targets and 27 NR2E3 target genes, 12 of which were common targets of both (Fig. 6). In addition, all potential target genes were cross-referenced against an RNA-Seq expression analysis of human macular RPE/choroid and macular retina from an AMD cohort of donor eyes. This cross-reference revealed 145 common genes. In particular, three genes (vascular endothelial growth factor receptor VEGFR1 (*FLT1*), protogenin (*PRTG*), and the synaptic adhesion molecule (*LRFN2*)) were significantly differentially expressed between normal RPE and Neo AMD RPE. *PRTG*, *LRFN2*, and the leucine zipper transcription factor, *BACH2* were significantly differentially expressed between neovascular AMD RPE and intermediate (AREDS3) RPE.

### Multimodal regulation of AMD gene networks by NRs, miRNAs, and Epigenetic factors

In addition to NHRs and miRNAs, a key component of gene regulation is epigenetics, and *Nr2e3* has been implicated in the *Ezh2* network^59^. In order to determine the epigenetic influence on miRNA and NHR target genes and gene networks potentially regulated by miRNAs and NHRs and associated with AMD. we compared the miRNA target genes against microarray data from P0 *Ezh2* ^*−*/*−*^ retinas as E18 and P0 time points have similar epigenetic effects (personal communication Dr. Chen). Although our studies were performed at E18, mouse gestation is 18–20 days and our prior unpublished work concurred that evaluating gene expression at E18 is quite similar to P0. In this cross-reference, we identified 7 genes and their 13 putative miRNAs as being significantly differentially expressed in *Ezh2* ^*−*/*−*^ compared to normal and also potential target genes of the miRNA identified in this study (Table 4). Furthermore, two of these genes (*Ell2* and *Entpd1*) are commonly modulated by the NHRs *Nr2e3* and *Rora* and are also differentially expressed in the RPE versus retina of AMD cohort.

**Table 4:** miRNAs and their potential target genes that are differentially expressed in rd7 vs B6 and Ezh2 vs B6.

Gene	miRNA	Expression TP
Pgm5	***mmu***-***miR***-***466a***-***5p***	E18, P6, P14, P30
	***mmu***-***miR***-***466d***-***5p***	E18, P6, P14, P30
	***mmu***-***miR***-***466e***-***5p***	E18, P6, P14, P30
	***mmu***-***miR***-***1187***	E18, P6, P14
***Ell2***	mmu-miR-1192	E18, P6, P14, P30
	mmu-miR-188-3p	E18
	mmu-miR-743b-3p	E18
Aspn	mmu-miR-369-3p	E18, P6, P14
**Jag1**	***mmu***-***miR***-***466f***-***3p***	E18, P6, P14, P30
	*mmu*-*miR*-*467f*	E18, P6, P14, P30
	*mmu*-*miR*-*467g*	E18, P6, P14, P30
	***mmu***-***miR***-***466d***-***3p***	E18, P6, P14
	*mmu*-*miR*-*710*	E18, P6, P14
Slc16a12	mmu-miR-188-3p	E18
Crhbp	***mmu***-***miR***-***466a***-***5p***	E18, P6, P14, P30
	***mmu***-***miR***-***466e***-***5p***	E18, P6, P14, P30
	***mmu***-***miR***-***1187***	E18, P6, P14
***Entpd1***	***mmu***-***miR***-***466a***-***5p***	E18, P6, P14, P30
	***mmu***-***miR***-***466e***-***5p***	E18, P6, P14, P30
	mmu-miR-327	E18
	***mmu***-***miR***-***1187***	E18, P6, P14

Genes and miRNAs regulated by NR2E3 are in bold; genes and miRNAs regulated by RORA are in italics, and genes and miRNAs regulated by NR2E3 and RORA are bold and italics. Minimum 2 fold change and p < 0.05.

## Discussion

This study is the first to evaluate multimodal regulation of gene expression by evaluating the combined impact of miRNAs, NHRs, and epigenetic factors. Recently, miRNAs have emerged as an important class of regulatory molecules that affect gene expression and in turn normal human development, function, and disease^15, 16^. miRNAs are endogenous non-coding RNA that regulate gene expression. Approximately 21,264 miRNAs have been identified to date and are classified in families based on sequence or the pre-miRNA structure^41, 48–92^. Over 400 miRNAs have been identified in the retina and function in the similar, parallel biological pathways in different tissues. This study reveals the synergistic impact of miRNAs, NHRs, and epigenetic factors on common biological networks in the normal and diseased state of the retina.

Several studies show that miRNA expression is subject to precise regulation that varies dramatically by cell type, developmental stage, environmental conditions, and disease pathology^14^. Currently, over 400 hundred miRNAs have been identified as key modulators of gene expression in the retina^14, 17, 93^. Here, we utilize a comprehensive strategy to identify and characterize multimodal nodes of gene regulation important in human retinal development and disease. We identified miRNAs that, along with the NHRs *Nr2e3* and *Rora* and the epigenetic factor *Ezh2*, play a synergistic role in modulating key biological networks affected in the complex human disease AMD. Eighteen overlapping gene networks were identified at E18 and P30 (Fig. 3A,B). These networks contained genes that regulate metabolism, immune response, development, and other cellular functions. We identified novel genes that are co-regulated by miRNAs, the nuclear hormone receptors *Nr2e3* and/or *Rora*. When comparing gene targets, NR2E3 and RORA have more targets in common at each time point (E18, 22 genes or P30, 11 genes) than for the same NHR at different time points (Fig. 3A,B). Furthermore, our study adds novel components to the list of *Nr2e3* and *Rora* targets identified thus far^42, 94^. We identified 4 miRNAs that are differentially expressed in the retina of affected *Nr2e3* ^*rd7*/*rd7*^ mice. These findings support the hypothesis that genetic variations strongly influence phenotypic outcome by perturbing gene regulation. We discovered 25 unique miRNAs with novel function in the retina. miR-466 and miR-1187, in particular, emerge as significant and regulators, sharing common target genes with *Nr2e3*, *Rora*, *Ezh2* that are also differentially expressed in AMD.

This study revealed several miRNAs and genes functioning in the developmental gene network. miR-466 was discovered in a recent study to identify miRNAs that target the Prospero homeobox 1 (*Prox1*) gene, a known regulator of retinal neurogenesis. More recent studies show *Prox1* influences inflammation-induced lymph-angiogenesis by regulating the expression of VEGF-C receptor. miR-466 in turn impacts lymph-angiogenesis by targeting *Prox1* 3′UTR sequence and suppressing the expression of the gene and therefore reducing lymphangiogenesis^95^. miR-1187 has no known function in the retina. Current studies show miR-1187 is down-regulated in a mouse model for acute liver failure and plays a role in hepatocyte apoptosis^96^. miR-1187 potential targets include Carboxypeptidase M (*Cpm*) a protein coding gene involved in monocyte and macrophage differentiation^97^, Ectonucleoside triphosphate diphosphohydrolase 1 (*Entpd1*) which functions in the hydrolyzes of phosphates into ATP, ADP, and AMP^98^, and Fraser extracellular matrix complex subunit 1(*Fras1*) that encodes for an extracellular matrix protein that regulates the epidermal-basement membrane adhesion as well as organogenesis in development^99^. *Alcam* codes for a transmembrane glycoprotein that belongs to the immunoglobulin family. *Alcam* has been implicated in the control of numerous developmental and pathological processes such as activation of T cells, melanoma cells, activation of monocytes, and has lately been used as a cancer stem cell marker^100, 101^. *Alcam* mutant retinas exhibit evaginated or invaginated regions with photoreceptor rosettes that resembled the retinal folds observed in some human retinopathies and also observed in the *Nr2e3* ^*rd7*/*rd7*^ retina^100^.

We identified seven putative miRNA target genes whose expression are also regulated by nuclear hormone receptors *Nr2e3*, *Rora*, and *Ezh2*: Phosphoglucomutase 5(*Pgm5*), Elongation factor for RNA polymerase II 2(*Ell2*), Asporin (*Aspn*), Jagged 1 (*Jag1*), and solute carrier family 16 member 12(*Slc16a12*), Corticotropin releasing hormone binding protein(*Crhbp*), Ectonucleoside Triphosphate Diphosphohydrolase (*Entpd1*). *Jag1* is the ubiquitously expressed, well known ligand of Notch and is part of a critical signaling pathway for the development of most tissues^102–104^. Our results add *Nr2e3* and *Ezh2* to the recently identified regulatory interplay between *Jag1* and 17beta-estradiol that is important in cancer and other tissue specific disorders including retinal diseases^105^. *Ell2*, codes for an RNA polymerase elongation factor and helps regulate the activity of the elongation complex. *Ell2* increases the rate of elongation during promoter dependent and independent transcription^106^. *Pgm5* is a phosphotransferase involved in the conversion of glucose^107^. In contrast the *Aspn* gene is involved in collagen mineralization and helps regulate chondrogenesis^108^. *Slc16a12* codes for a transmembrane transporter and plays a role in monocarboxylic acid transport. Mutations in *Slc16a12* are associated with juvenile cataracts^109^. Lastly, *Crhbp* is a corticotropin hormone and plays a role in development of the optic cup^110^. Our study connects several known, important genes and biological processes with novel miRNAs in normal and diseased retina.

Age-related macular degeneration is a leading cause of blindness in the world. In this study we discovered three key known AMD genes, Fms related tyrosine kinase 1(*Flt1*), the receptor for vascular endothelial growth factor (VEGF) (putative miR1192 target), ATP binding cassette subfamily A member 1 (*Abca1*) (putative miR673-5p target), and Activated leukocyte cell adhesion molecule(*Alcam*) (putative miR188-3p target) are regulated by *Nr2e3* and *Rora* and their potential corresponding miRNAS that are differentially expressed in *Nr2e3* ^*rd7*/*rd7*^ retinas. *Flt1*, a receptor tyrosine kinase, functions in the VEGF pathway, is predominantly expressed in endothelial cells, and is the target for transducing signal in VEGF treatment^111^. Moreover, our group previously determined *Flt1* is a target of *Nr2e3*, and demonstrated genetic variants in *Flt1* in four ethnically diverse patient populations associated with AMD^112^. *Abca1* is an ATP transporter that is expressed in the retina as well as in the RPE^113^. Alleles of the *Abca1* gene are associated with a reduction in the levels of HDL contributing to the formation of drusen accumulation in AMD^114^. Variants in *Abca1* have also been shown to be associated with risk of AMD^115^. In addition, the miRNAs mir466 and mir1186 have 5 potential target genes that are differentially expressed in AMD patients compared to unaffected individuals: lymphocyte cytosolic protein1 (*Lcp1*), NADPH oxidase 4 (*Nox 4*) regulator of G-protein signaling (*Rgs5*), Dystonin (*Dst*), and *Cpm*.

Recent studies show an important role for the *Nr2e3* in cancer^116–118^. Interestingly, in this study we identified several miRNAs that are differentially expressed in *rd7* and play a significant role in cancer. The *rd7* model does not develop cancer, however it is well documented that *rd7* mice have a defect in cell proliferation^41, 119^. Consistent with reports that miRNAs are predominantly downregulated in cancer tumors^120^, our studies show that 85% of the miRNAs that are differentially expressed in *rd7* are downregulated. Many of these miRNAs are also subject to epigenetic regulation and are located within CpG islands and regulated by methylation^121^. Interestingly, our IPA analysis also revealed the miRNA miR125b-5p is associated with diabetes and cancer (Table 3). A study in breast cancer lines demonstrated a direct link between the methylation status of the cancer miRNA promoters and the development of cancer^122^. These findings further substantiate an important role for nuclear receptors such as Nr2e3 in broader-impact diseases such as cancer. Our future studies will further explore the role of Nr2e3 in cancer and regulation of metastasis via miRNAs and NHRs.

While numerous studies show the significant singular impact that NHRs, miRNAs, and epigenetics have on mammalian development and human disease, *in vivo*, the process is dynamic and complex involving multiple factors simultaneously converging for singular biological events. This study is the first to demonstrate the synergistic and combined impact of multiple factors on gene expression in the developing and mature retina and its impact on human disease. While these studies were conducted in the retina, the genes and microRNAs identified here are not unique to the retina or even the CNS. The findings of this study will provide a better understanding of normal development, genomic signatures modulating complex human retinal disease, and valuable tools for better prognostics and development of improved therapies. Future studies will explore developing combination therapies to combat human retinal diseases such as AMD.

## Materials and Methods

### Ethics Statement

All animals were bred and maintained under standard conditions at The University of Nebraska Medical Center or Schepens Eye Research Institute research vivarium in accordance with protocol numbers 04086, approved by the Animal Care and Use Committee at the University of Nebraska Medical Center, and 3980417 and 4060617 at Schepens Eye Research Institute. Mice were housed in microisolator cages and provided food and water ad libitum. The University of Nebraska Medical Center is in compliance with the NIH policy on the use of animals in research (Animal Welfare Act P.L. 89–544, as amended by P.L. 91–579 and P.L. 94–279) as well as the Guide for the Care and Use of Laboratory Animals, NIH Publication No. 86–23. All experiments were performed in accordance with relevant guidelines and regulations.

### Availability of Data and Materials

The miRNA dataset supporting the conclusion of this article has been made MIAME compliant and is publicly available through the Gene Expression Omnibus (GEO) database under the series record GSE91029, and the prior microarray used to cross reference miRNA targets are available under the series record GSE24512. AMD data used as cross reference in this study is in preparation for publication.

### Microarray Analysis

MicroRNA microarrays were performed using mirVana 2-color microRNA array chips hybridized to 100 nanogram (ng) of total miRNA from 10 pool retinas for each time point from *Nr2e3* ^*rd7*/*rd7*^ and C57BL6/J (B6) control retinas extracted at E18, P6, P14, or P30. Samples were hybridized to mirVana miRNA chips followed by normalization using Lowess normalization and generation of scatter plots using GenePix to determine the strong data points. Chips were normalized using both whole chip normalization and control spot normalization. Heat map of raw data is found in Supplemental Figure 1. Differential miRNA expression was determined utilizing a merged data set of both normalization techniques and the following criteria: (1) >20% spots showing >2-fold change; (2) P < 0.001 for log-ratio variation; (3) exclusion of array spots with >50% missing values. Additional analysis of differentially expressed miRNAs was performed using BRB Array Tools for Excel, as well as the Stanford Statistical Analysis of Microarrays (SAM) plug-in for Excel. miRNA expression data were input into SAM using a false discovery rate (FDR) of <0.05. Heat map for the microarray analysis with a hierarchical cluster analysis has been previously published^91^. Heat map depicting differential expression of miRNA as observed by miRNA array. Values represent median intensity values for the respective label (cy5/cy3). Color-coding of heat map is relative to percentile higher expression values represented in orange and lower expression values in dark indigo (Supplemental Figure 1). All differentially expressed miRNAs were subsequently evaluated against the mirBase to determine all potential target genes for each miRNA. Each miRNA can have up to 700–1500 potential target genes.

A cross-comparison was made between the miRNA array data (differentially expressed miRNAs) with microarray data (gene expression >2 fold change). RNA was isolated at E18.5, P6, P14, and P30 from *Nr2e3* ^*rd7*/*rd7*^ retinas as previously described^42^. Eyes were enucleated from 7 different animals (at each time point) and placed in phosphate buffered saline (PBS) on ice. Retinas were dissected using a stereomicroscope (Zeiss SV11) and RNA was isolated by TRIzol® extraction. RNA was hybridized to Mouse 420A 2.0 (Affymetrix, Santa Clara, CA) chips by the UNMC Microarray Core Facility according to manufacturer specifications (Affymetrix, Santa Clara, CA). Data quality was assessed using the affyPLM package for the R programming language. Consistency of expression levels was confirmed by validation across multiple redundant probe sets. Differential expression analysis was performed using the Linear Models for Microarray Analysis portion of Bioconductor. Genes found to be differentially expressed for each pair wise comparison using an FDR-adjusted p-value of 0.001 and at least a 2-fold change were combined and used to perform clustering analysis. A self-organizing map (SOM) clustering algorithm was applied to genes showing significant expression differences as judged by mean log2 intensity per strain. The gap statistic was used to estimate the optimal number of clusters.

### Quantitative RT-PCR

Each gene and miRNA identified through microarray studies were validated through quantitative real-time PCR (qRT-PCR). Real time PCR was performed as previously described^42^ to confirm differential expression observed in the microarray data. RNA was isolated using the samples from the microarray analysis as well as additional individual samples from mice for each strain (total n = 7 per sample type). Two µg of total RNA was reverse transcribed using Retroscript (Ambion). The cDNA samples were diluted 1:100 and real time PCR was performed in triplicates for each primer using Sybr Green PCR master mix (Thermo fisher). Reactions were quantified using an ABI Step One Plus Real Time PCR and analyzed with the corresponding software. Relative expression levels were determined by normalizing cycle thresholds values to the amount of β-actin expressed (1000/2^Ct gene − Ct β-actin) Statistical significance of differential expression was assessed using a T-test and p < 0.05.

### Pathway Generation and Analysis

miRNA and microarray target genes were analyzed using Ingenuity Pathway Analysis (IPA, Ingenuity Systems, www.ingenuity.com). Gene identifiers and statistically significant expression values were uploaded into Ingenuity. Default cutoffs were set to identify genes whose expression was significantly differentially regulated and overlaid onto a global molecular network developed from information contained in the Ingenuity Pathways Knowledge Base. Networks were algorithmically generated based on their connectivity. Genes or gene products in the networks are represented as nodes, and the biological relationship between two nodes is represented as an edge (line). All edges are supported by at least 1 reference from the literature, from a textbook, or from canonical information stored in the Ingenuity Pathways Knowledge Base. Nodes are displayed using various shapes that represent the functional class of the gene product.

### Chromatin Immunoprecipitation

Putative miRNA target genes were analyzed for nuclear receptor binding sites using: 1) classic binding sites of *Nr2e3* (AAGTCA_(n1-4)_AAGTCA) or *Rora* ([AGT][TA][AT][TA]C[AT]AGGTCA); 2) general nuclear hormone receptor response elements as determined algorithmically by NUBIscan^123^. Binding sites were selected at a maximum of 100 kilobases (kb) upstream of each gene’s start site to intron 1. Chromatin immunoprecipitation was performed as described previously^42^. Briefly, retinas were dissected from P30 C57Bl/6J mice. A total of 8–10 retinas were used per Chip reaction and placed into 400 uL of a PI solution (phosphate buffered saline (PBS) and protease inhibitor cocktail from Roche). Chilled tissue was dissociated and homogenized and cross-linked in 37% formaldehyde for a period of 60 minutes at room temperature in a rotating platform. 1 M glycine was added (final concentration 0.125 mM) and later rinsed with the phosphatase inhibitor (PI) solution in PBS (Thermo, cat#78430). Sonication was performed using a Qsonica Ultrasonic Processor amplitude 50%, 10 pulses x 30 with 1-second pauses between pulses. Immunoprecipitation was performed overnight using 1 μg of NR2E3 antibody at 4 °C on a rotating platform. The NR2E3 antibody used in this experiment had been previously generated in the laboratory^43^ and validated^42^. For a negative control goat IgG antibody was used and the input (control) was not incubated with antibody. Immunoprecipitated samples were resuspended with Protein G dynabeads (Invitrogen, cat#10003D) and a series of washes using wash buffer #1 (Tris, EDTA 0.5 M, Triton, NaCl 5 M) and wash buffer #2 (Tris, EDTA 0.5 M, NP-40, LiCl) were performed at room temperature. Samples were reversed cross-inked with NaCl (200 mM final concentration). Quantitative RT-PCR was performed using 1 ul of 1:100 dilution (input) and 1:10 dilution (samples and immunoglobulin G (IgG) control) using conditions described previously^36^. All sample data was normalized to IgG control.

### Comparison to Human RNA-Seq Data

Resulting mouse data was compared to differentially expressed genes found in the Utah human donor eye AMD repository and RNA-Seq dataset (personal communication DeAngelis lab) from macular neural retina and macular RPE/choroid. Cohort is comprised of Caucasians over the age of 60 including representatives of both intermediate and neovascular subtypes with age matched controls.

## Electronic supplementary material

Supplementary Information
