# Supplementary material for: Multimodal Regulation Orchestrates Normal and Complex Disease States in the Retina
Source: Sci Rep. 2017 Apr 6;7:690. doi: 10.1038/s41598-017-00788-3 (PMC5429617; doi:10.1038/s41598-017-00788-3)
Supplement: Supplementary file 1 — Supplementary Information [file 41598_2017_788_MOESM1_ESM.pdf]

Multimodal Regulation Orchestrates Normal and Complex Disease States in the Retina

Olivares, AM[1]. Jelcick, AS[2]. Reinecke, J[2]. Leehy, B[2]. Haider, A[1], Morrison, MA[3], Cheng, L[1]. Chen, DF.[1], DeAngelis, MM[3]. Haider, NB[1]*.

[1]Schepens Eye Research Institute, Massachusetts Eye and Ear Infirmary, Department of Ophthalmology, Harvard Medical School, Boston, MA, United States of America

[2]Genetics, Cell Biology, and Anatomy, University of Nebraska Medical Center, Omaha, Nebraska, United States of America

[3]Ophthalmology and Visual Sciences, John A. Moran Eye Center, University of Utah School of Medicine, Salt Lake City, Utah, United States of America

Supplemental Figure 1. Heat map of raw miRNA data. Color-coding of heat map is relative to percentile higher expression values represented in orange and lower expression values in dark indigo. X axis indicates individual samples (B6 or *rd7* for E18, P6, P14, or P30) in triplicate; Y axis indicates individual miRNAs.

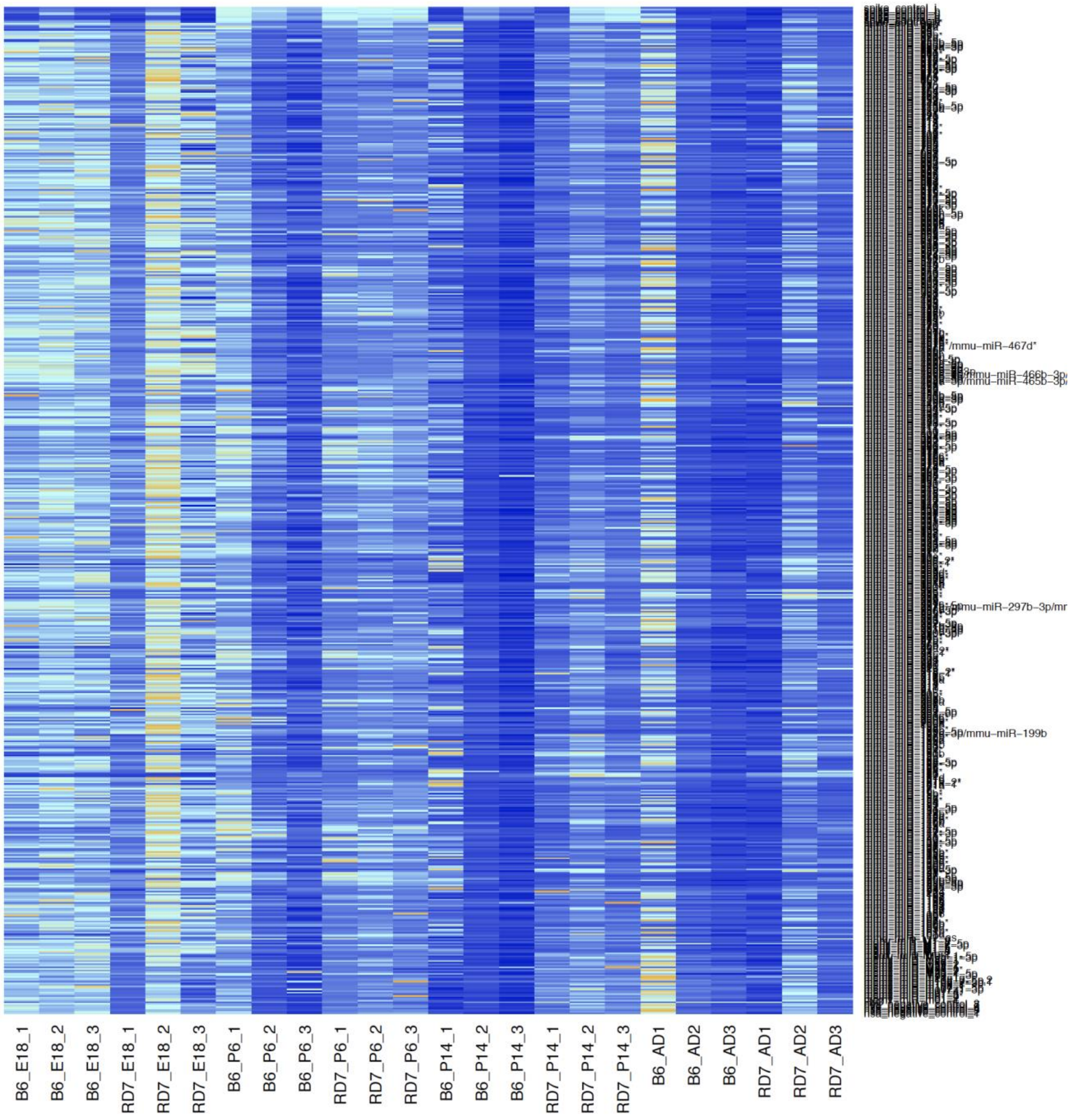

Supplemental Table 1. Potential miRNA target genes that are differentially expressed in B6 vs *rd7* microarray. Cross-reference of miRNA potential targets with E18 or P30 microarray data (GSE24512) of differentially expressed genes.

ID	Gene Name
Abca9	ATP binding cassette subfamily A member 9
Abca1	ATP binding cassette subfamily A member 1
Abcc9	ATP binding cassette subfamily C member 9
Acer2	Alkaline ceramidase 2
Acpp	Acid phosphatase, prostate
Afap1l2	Actin filament associated protein 1 like 2
Alcam	Activated leukocyte cell adhesion molecule
Antxr2	Anthrax toxin receptor 2
Arap2	ArfGAP with RhoGAP domain, ankyrin repeat and PH domain 2
Arg1	Arginase 1
Arhgap36	Rho GTPase activating protein 36
Arhgef3	Rho guanine nucleotide exchange factor 3
Aspn	Asporin
B3gnt9	UDP-GlcNAc:betaGal beta-1,3-N-acetylglucosaminyltransferase 9
Bach2	BTB domain and CNC homolog 2
Bmf	Bcl2 modifying factor
Bmp7	Bone morphogenetic protein 7
Bnc2	Basonuclin 2
Boc	BOC cell adhesion associated, oncogene regulated
Casp12	caspase 12
Cast	calpastatin
Cav3	caveolin 3
Ccbe1	collagen and calcium binding EGF domains 1
Ccnd2	cyclin D2
Cd248	CD248 molecule
Cd36	CD36 molecule
Cd44	CD44 molecule (Indian blood group)
Cd55	CD55 molecule (Cromer blood group)
Cgnl1	Cingulin-like 1
Col18a1	Collagen type XVIII alpha 1
Col25a1	Collagen type XXV alpha 1
Cpm	Carboxypeptidase M
Crhbp	Corticotropin releasing hormone binding protein
Crim1	Cysteine rich transmembrane BMP regulator 1 (chordin-like)

Cspg4	Chondroitin sulfate proteoglycan 4
Cxcl12	C-X-C motif chemokine ligand 12
Dek	DEK proto-oncogene
Dennd2c	DENN domain containing 2C
Dlk1	Delta-like 1 homolog (Drosophila)
Dmrta2	DMRT like family A2
Dpt	Dermatopontin
Dsc2	Desmocollin 2
Dsg2	Desmoglein 2
Dst	Dystonin
Eef2k	Eukaryotic elongation factor 2 kinase
Egfl6	EGF like domain multiple 6
Egfr	Epidermal growth factor receptor
Egln3	Egl-9 family hypoxia-inducible factor 3
Ehd4	EH domain containing 4
Ehf	ETS homologous factor
Elk3	ELK3, ETS transcription factor
Ell2	Elongation factor for RNA polymerase II 2
Elovl7	ELOVL fatty acid elongase 7
Entpd1	Ectonucleoside triphosphate diphosphohydrolase 1
Ets1	ETS proto-oncogene 1, transcription factor
Fam101b	Family with sequence similarity 101 member B
Fam114a1	Family with sequence similarity 114 member A1
Fam174b	Family with sequence similarity 174 member B
Fam46a	Family with sequence similarity 46 member A
Fam84a	Family with sequence similarity 84 member A
Fbn2	Fibrillin 2
Fbxl7	F-box and leucine-rich repeat protein 7
Fgfr2	Fibroblast growth factor receptor 2
Fgl2	Fibrinogen like 2
Fibin	Fin bud initiation factor homolog (zebrafish)
Flrt2	Fibronectin leucine rich transmembrane protein 2
Flt1	Fms related tyrosine kinase 1
Fndc1	Fibronectin type III domain containing 1
Fndc3b	Fibronectin type III domain containing 3B
Fosl2	FOS like antigen 2
Foxc1	Forkhead box C1
Fras1	Fraser extracellular matrix complex subunit 1
Frk	Fyn related Src family tyrosine kinase
Fzd4	Frizzled class receptor 4
Fzd6	Frizzled class receptor 6

Gatm	Glycine amidinotransferase
Gbp7	Guanylate binding protein 7
Grhl2	Grainyhead like transcription factor 2
Hapln1	Hyaluronan and proteoglycan link protein 1
Has2	Hyaluronan synthase 2
Hmcn1	Hemicentin 1
Ifi202b	Interferon activated gene 202B
Il13ra1	Interleukin 13 receptor subunit alpha 1
Itgb3	Integrin subunit beta 3
Itpripl2	Inositol 1,4,5-trisphosphate receptor interacting protein-like 2
Jade2	Jade family PHD finger 2
Jag1	Jagged 1
Kcnq5	Potassium voltage-gated channel subfamily Q member 5
Kdelc2	KDEL motif containing 2
Lamc1	Laminin subunit gamma 1
Lamp2	Lysosomal associated membrane protein 2
Lcp1	Lymphocyte cytosolic protein 1
Lmna	Lamin A/C
Lmo7	LIM domain 7
Lpar1	Lysophosphatidic acid receptor 1
Lpp	LIM domain containing preferred translocation partner in lipoma
Lrfn2	Leucine rich repeat and fibronectin type III domain containing 2
Lrig1	Leucine-rich repeats and immunoglobulin like domains 1
Lyz1	Lysozyme
Matn2	Matrilin 2
Mef2c	Myocyte enhancer factor 2C
Mgll	Monoglyceride lipase
Msrb3	Methionine sulfoxide reductase B3
Muc4	Mucin 4
Mylk4	Myosin light chain kinase family member 4
Myot	Myotilin
Myrf	Myelin regulatory factor
Nebl	Nebulette
Nox4	NADPH oxidase 4
Npr3	Natriuretic peptide receptor 3
Nrk	Nik related kinase
Nrp2	Neuropilin 2
Ntrk2	Neurotrophic receptor tyrosine kinase 2
Osr1	Odd-skipped related transciption factor 1
Ostf1	Osteoclast stimulating factor 1
Otx1	Orthodenticle homeobox 1

Papss2	3'-phosphoadenosine 5'-phosphosulfate synthase 2
Pdlim7	PDZ and LIM domain 7
Peg3	Paternally expressed 3
Pgm5	Phosphoglucomutase 5
Piezo2	Piezo type mechanosensitive ion channel component 2
Ppargc1a	PPARG coactivator 1 alpha
Ppfibp2	PPFIA binding protein 2
Ppp1r3b	Protein phosphatase 1 regulatory subunit 3B
Prdm1	PR domain 1
Prdm16	PR domain 16
Prickle1	Prickle planar cell polarity protein 1
Prtg	Protogenin
Ptgfrn	Prostaglandin F2 receptor inhibitor
Ptprb	Protein tyrosine phosphatase, receptor type B
Rab27a	RAB27A, member RAS oncogene family
Rbms1	RNA binding motif single stranded interacting protein 1
Rgs5	Regulator of G-protein signaling 5
Rspo1	R-spondin 1
Ryr1	Ryanodine receptor 1
Scara5	Scavenger receptor class A member 5
Sdpr	Serum deprivation response
Sema3c	Semaphorin 3C
Sema3d	Semaphorin 3D
Sgms1	Sphingomyelin synthase 1
Sgms2	Sphingomyelin synthase 2
She	Src homology 2 domain containing E
Slc16a12	Solute carrier family 16 member 12
Slc7a10	Solute carrier family 7 member 10
Slc7a8	Solute carrier family 7 member 8
Smoc2	SPARC related modular calcium binding 2
Smpx	Small muscle protein, X-linked
Sspn	Sarcospan
Stard8	StAR related lipid transfer domain containing 8
Stat6	Signal transducer and activator of transcription 6
Sulf1	Sulfatase 1
Tbx18	T-box 18
Tbx22	T-box 22
Tek	TEK receptor tyrosine kinase
Tfcp2l1	Transcription factor CP2-like 1
Tgfbr2	Transforming growth factor beta receptor 2
Tgfbr3	Transforming growth factor beta receptor 3

Thbs1	Thrombospondin 1
Thbs2	Thrombospondin 2
Tmem164	Transmembrane protein 164
Tnnt2	Troponin T2, cardiac type
Trdn	Triadin
Trp63	Tumor protein p63
Ucp2	Uncoupling protein 2
Unc45b	Unc-45 myosin chaperone B
Vamp5	Vesicle associated membrane protein 5
Vcan	Versican
Wnt4	Wnt family member 4
